# Novel Anti-Interleukin-1β Therapy Preserves Retinal Integrity: A Longitudinal Investigation Using OCT Imaging and Automated Retinal Segmentation in Small Rodents

**DOI:** 10.3389/fphar.2020.00296

**Published:** 2020-03-12

**Authors:** Diane N. Sayah, Tianwei E. Zhou, Samy Omri, Javier Mazzaferri, Christiane Quiniou, Maëlle Wirth, France Côté, Rabah Dabouz, Michel Desjarlais, Santiago Costantino, Sylvain Chemtob

**Affiliations:** ^1^Hopital Maisonneuve-Rosemont Research Center, Montreal, QC, Canada; ^2^Department of Ophthalmology, Faculty of Medicine, Université de Montréal, Montreal, QC, Canada; ^3^Faculty of Medicine, McGill University, Montreal, QC, Canada; ^4^Department of Pediatrics, Centre Hospitalier Universitaire Sainte-Justine Research Center, Université de Montréal, Montreal, QC, Canada

**Keywords:** rytvela, kineret, anti-interleukin-1β, therapy, oxygen-induced retinopathy, retina, optical coherence tomography, automated segmentation

## Abstract

Retinopathy of prematurity (ROP) is the leading cause of blindness in neonates. Inflammation, in particular interleukin-1β (IL-1β), is increased in early stages of the disorder, and contributes to inner and outer retinal vasoobliteration in the oxygen-induced retinopathy (OIR) model of ROP. A small peptide antagonist of IL-1 receptor, composed of the amino acid sequence, rytvela, has been shown to exert beneficial anti-inflammatory effects without compromising immunovigilance-related NF-κB in reproductive tissues. We conducted a longitudinal study to determine the efficacy of “rytvela” in preserving the integrity of the retina in OIR model, using optical coherence tomography (OCT) which provides high-resolution cross-sectional imaging of ocular structures *in vivo*. Sprague–Dawley rats subjected to OIR and treated or not with “rytvela” were compared to IL-1 receptor antagonist (Kineret). OCT imaging and custom automated segmentation algorithm used to measure retinal thickness (RT) were obtained at P14 and P30; gold-standard immunohistochemistry (IHC) was used to confirm retinal anatomical changes. OCT revealed significant retinal thinning in untreated animals by P30, confirmed by IHC; these changes were coherently associated with increased apoptosis. Both rytvela and Kineret subsided apoptosis and preserved RT. As anticipated, Kineret diminished both SAPK/JNK and NF-κB axes, whereas rytvela selectively abated the former which resulted in preserved monocyte phagocytic function. Altogether, OCT imaging with automated segmentation is a reliable non-invasive approach to study longitudinally retinal pathology in small animal models of retinopathy.

## Introduction

Retinopathy of prematurity (ROP) is the leading cause of severe visual impairment and blindness in neonates and young children in the western world. In the early stages of the disease, pro-inflammatory IL-1β increases markedly, resulting in microvascular decay which culminates in intravitreal neovascularization predisposing to retinal detachment (Penn et al., 1994; Rivera et al., 2013; Zhou et al., 2016). Recently, rytvela, an Interleukin-1 receptor (IL-1R) allosteric modulator, was shown to be effective in preserving retinal microvascular integrity in ischemic retinopathy induced by postnatal hyperoxia (Rivera et al., 2013) and antenatal inflammation (Beaudry-Richard et al., 2018). Akin to other biologics, IL-1 receptor antagonist (commercialized as Kineret) broadly diminishes immunologic functions of IL-1β, thus increasing the likelihood of serious infections, a major concern for relatively immune incompetent immature neonates. Whereas biased signaling modulation by rytvela targets MAPK and Rho GTPase pathways, while desirably preserving immunovigilant-related NF-κB (Nadeau-Vallee et al., 2015).

Optical coherence tomography (OCT) is now widely used in the clinical setting. Based on low-coherence interferometry, Spectral Domain (SD)-OCT provides high-resolution cross-sectional imaging of ocular structures, permitting the non-invasive observation of retinal layers *in vivo* (Huang et al., 1991). We recently adapted OCT modality to laboratory animals by developing a protocol to efficiently acquire OCT images in small rodents (Zhou et al., 2017). OCT allows to conduct longitudinal studies, accounts for inter-individual variability, and reduces the number of animals required, leading to more robust interpretation of therapeutic pre-clinical trials. In addition, automated segmentation delineates regions in an image using computerized algorithms and allows for more rapid processing of data. In this context, robust segmentation algorithms identify characteristics of a tissue, provide measurements of its dimensions, and compared to manual tracing are efficient, reliable, and objective, as we have demonstrated for human eyes (Beaton et al., 2015; Mazzaferri et al., 2017); OCT and these algorithms can be adapted for small rodents as used herein. Using the oxygen-induced retinopathy (OIR) model of ROP in small rodents, we aim to compare in a longitudinal study using OCT imaging coupled with automatic segmentation the efficacy of rytvela with that of Kineret in preventing retinal damage that follows the OIR-induced vasoobliteration.

## Materials and Methods

This study was carried out in accordance with the principles of the Basel Declaration and Hôpital Maisonneuve-Rosemont Animal Care Committee approved protocols (authorizations 2017-1320, 2016-AV-004), and is adherent to the International Association of Veterinary Editors guidelines.

### Oxygen-Induced Retinopathy Model

Newborn Sprague–Dawley rats (Charles River, St-Constant, QC, Canada) were placed under an oxygen concentration protocol cycling between 50 ± 1 and 10 ± 1% every 24 h from postnatal day (P) 0 to P14 (OIR/ROP) (Penn et al., 1994). On P14 rats were returned to room air. This model is characterized by retinal vascular decay (Penn et al., 1994; Rivera et al., 2017) followed by hypoxia-driven inner retinal neovascularization which develops between P14 and P18. Control rats (NOR) were maintained in room air (21% O_2_). Rats (*n* = 4/group), exposed or not to OIR, were randomly assigned to receive from P0 to P14 either twice-daily intraperitoneal injections of rytvela [1 mg/kg; custom synthesized by Elim Pharmaceuticals (>95% purity)], Kineret (20 mg/kg, Biovitrum), or no treatment. The posology of rytvela was based on reported efficacy (Quiniou et al., 2008) and further supported in subsequent studies (Nadeau-Vallee et al., 2015; Beaudry-Richard et al., 2018; Geranurimi et al., 2019).

### Longitudinal Study Design

Twenty-four newborn Sprague–Dawley rats were divided equally into six groups. Twelve rats were placed in conditions to develop OIR as explained previously. The healthy control group comprised twelve other rats. In both the OIR and healthy groups, four rats were treated with rytvela, four rats were treated with the Kineret, and four rats received no treatment. Imaging took place at two time points, P14 and P30. On P30, all animals were then sacrificed and eyes were immediately collected and prepared for histological analysis.

### Optical Coherence Tomography Imaging

Spectral domain optical coherence tomography (Spectralis OCT, Heidelberg Engineering) imaging was carried out on all rats at P14 and P30 after the careful dosing injection of ketamine-xylazine for anesthesia (Zhou et al., 2017). The anesthetized rats were placed on a plastic horizontal panel in front of the OCT for imaging. The instillation of dilating drops, tropicamide (Mydriacyl) 1.0%, in one or both eyes was done. Additional steps to ensure high-quality OCT images included: (1) placing anesthetized rats on a warming pad (35–37°C) during image acquisition and until they regained full consciousness as advised by the Canadian Council on Animal Care; (2) frequent hydration (every 30 s) of the cornea with artificial tears to restore crispness of fundus and OCT images; and (3) lubricating ointment (Tear Gel, Alcon) application to the unexamined eye during OCT acquisition and to the examined eye immediately post-acquisition.

#### OCT Parameters

Volume scans of 15°× 5° (7 B-scans 240 microns apart, ART 100 frames including 768 A-scans) were taken in the right eye, by convention. If the imaging was rendered difficult due to rapid breathing or movement that disrupted the eye-tracker, the experimenter held the rat’s head in place and applied light pressure to reduce motion and permit proper OCT imaging. All OCT scans were obtained at the temporal side of the optic nerve (equivalent to position of human macula). During acquisition, image quality was determined based on the Spectralis image quality signal as seen on the OCT monitor, as well as subjective appreciation of the resolution of the B-scan and clear visualization of the layers of interest by the examiner.

### Automatic Segmentation of OCT Images

**FIGURE 1:**
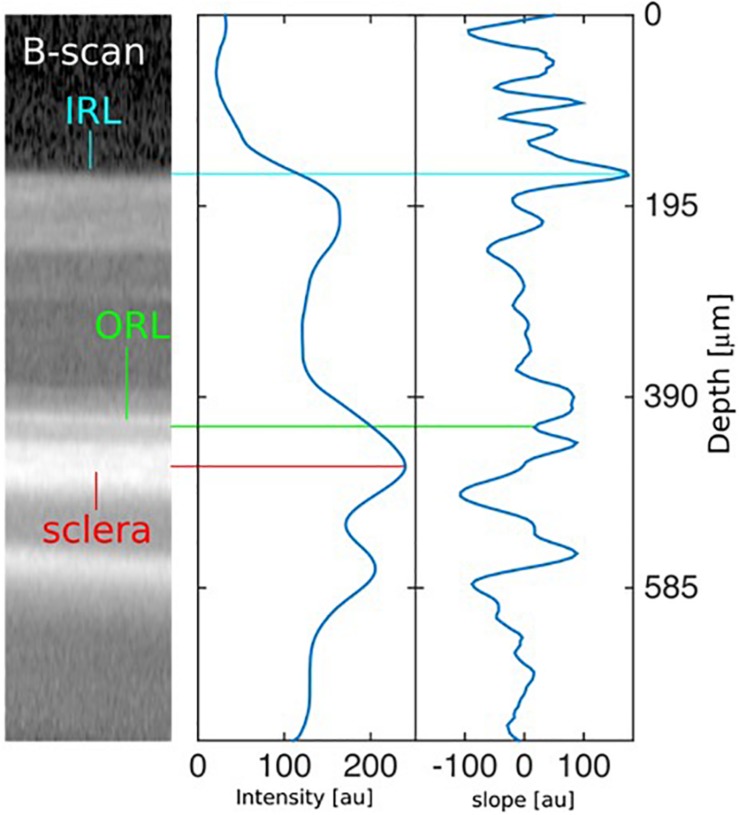
Description of automated segmentation algorithm. Left: Identification of the sclera, the outermost retinal layer (ORL), and the innermost retinal layer (IRL) in a section of a typical rodent eye B-scan. Center: Intensity profile along the center A-scan of the image at the left, averaged with a Gaussian filter (σ = 20 μm). The absolute maximum signals the location of the sclera. Right: First derivative (slope) of the intensity profile along the center A-scan of the image at the left. The first valley above the sclera indicates the ORL, and the highest peak more than 120 μm above it indicates the IRL. The slope is computed after a Gaussian smoothing operation with σ = 2 μm.

Image processing for measuring the thickness of the retina in OCT B-scans of rodent eyes was carried out using a custom-made fully automated segmentation algorithm with Matlab (The Mathworks, Inc.). Essentially, the algorithm segments two layers sequentially: the outermost retinal layer (ORL) and the innermost layer of the retina (IRL) as seen in Figure 1. The sclera being the brightest object in the B-scan, this feature was used to coarsely localize it in the image. After smoothing the image with a Gaussian average filter (σ = 20 μm), we located the center of the scleral layer as the absolute intensity maximum in each A-scan (Figure 1, center panel). Taking this position as a reference, we located the ORL as the first valley of the intensity slope just above the center of the sclera. Finally, the IRL was obtained as the highest intensity slope peak more than 120 μm above the ORL. The intensity slope was computed after a Gaussian smoothing operation using a filter of σ = 2 μm.

The retinal thickness (RT) was computed as the distance between the IRL and the ORL. This procedure was performed on every A-scan of each B-scan of each OCT volume scan. The mean thicknesses and their standard deviations were computed for each B-scan, using the results of all A-scans. After discarding B-scans where the percentage error was bigger than 25%, the mean thickness for each map was computed as the weighted average along B-scans, using the inverse of the standard deviation as weight. The final uncertainty of the thickness was computed as the standard error among B-scans.

### Ocular Tissue Preparation

Animals were perfused with phosphate buffered saline and 4% paraformaldehyde. For histology sections, eyes were immediately collected, dehydrated by alcohol, and embedded in paraffin. Sagittal sections of 5 μm were cut by microtome (Leica, RM 2145). Eyes for cryo-preparation were further fixed in 4% paraformaldehyde overnight. Posterior eyecups were frozen in optimal cutting temperature medium. Samples were then cut into 10 μm-thick sagittal sections (Microm, HM500O) and processed for IHC.

### Cell Culture

The murine macrophage cell line J774 and RAW264.7, purchased from ATCC, were cultured in Dulbecco’s Modified Eagle’s Medium (DMEM) (Thermo Fisher Scientific, 11995-065) supplemented with 10% fetal bovine serum (FBS) and 1% penicillin/streptomycin (respectively, 085-150 and 450-201-EL, Wisent Bioproducts).

### Phagocytosis Assay Preparation

Mouse macrophages (Raw 264.7 and J774) (100,000 cells) were pre-incubated with rytvela (1 μM), SC-514 (2 μM), or Kineret (1 mg/ml) for 30 min and incubated with IL-1β (100 ng/ml) for 4–24 h. Phagocytosis was determined with the Vybrant Phagocytosis Assay kit from Thermo Scientific (Waltham, MA, United States) according to manufacturer’s instructions. Briefly, medium was removed at 4 or 24 h after IL-1β incubation and replaced with Fluorescein-labeled *Escherichia coli* K-12 BioParticles resuspended in HBSS. Two hours later, bioparticles were removed and the signal was quenched by exposing cells to trypan blue for 1 min. Fluorescence intensity was read using 480 nm excitation and 520 nm emission using ClarioStar microplate reader (BMG LabTech; Champigny-sur-Marne, France). The same procedure was repeated using Kineret and SC-514, an inhibitor of NF-κB and results were compared with those obtained with rytvela.

### Immunofluorescence Staining

J774 macrophages were seeded overnight in DMEM containing 10% FBS and 1% penicillin/streptomycin, on round 15 mm cover glass. J774 were pre-incubated with rytvela (1 μM), for 30 min and incubated with IL-1β (100 ng/ml) for 4 h. Then medium was removed and Fluorescein-labeled *Escherichia coli* K-12 BioParticles mix was added with HBSS. Two hours later, cells were washed twice with PBS for 5 min, fixed in 4% PFA for 30 min, and permeabilized in 1.0% Triton X100 and blocked in 10% FBS 1 h. Cells were counterstained with Rhodamine Phalloidin (1:500, 1 h) (Santa Cruz Biotechnology, R415) and DAPI (1:5000; 5 min) (Sigma–Aldrich, D9542) to evidence cell-contour and cell-nuclei. Phagocytosis efficiency was assessed using a confocal microscope (Zeiss, LSM 510).

### Statistical Analysis

Results for RT are presented as means ± standard error of the mean. One-way ANOVA with significance (α = 0.05) was used for processing data. Bonferroni *post hoc* analysis was used to calculate statistical significance between groups. The graphs showing phagocytosis results were generated using Graph Prism 8. One-way ANOVA with significance (α = 0.05) was used for comparing experiment data.

## Results

### Early Anti-IL-1 β Therapy Preserves Retinal Thickness in OIR Animals

**FIGURE 2:**
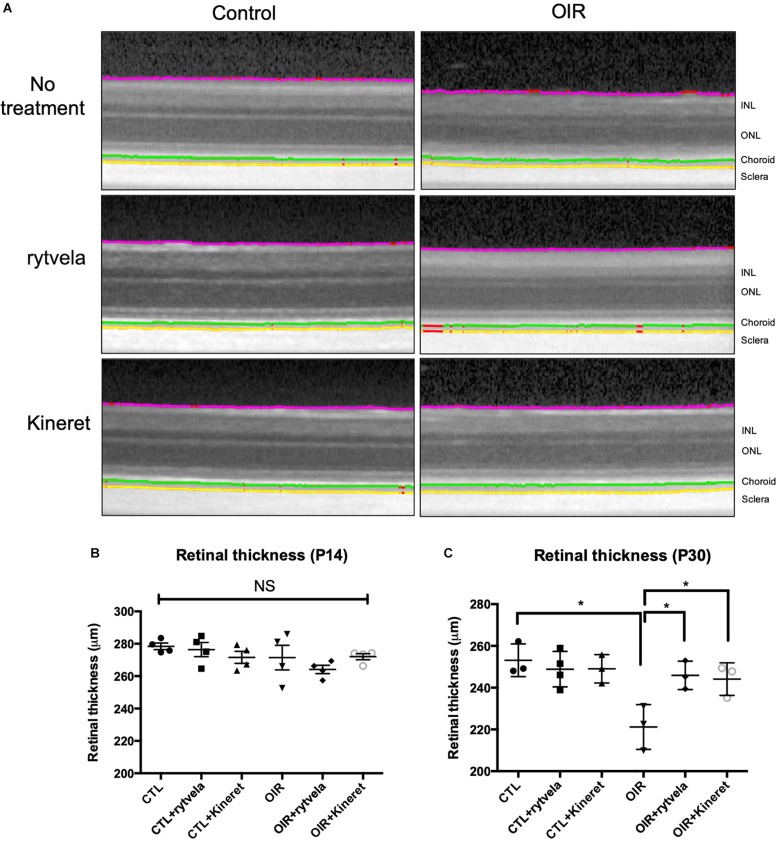
Early anti-IL-1β therapy preserves retinal integrity in OIR subjects. **(A)** Original B-scans overlaid with the innermost layer of the retina (purple), the inner choroid limit (green), and the choroid-sclera interface (yellow) are shown for each sub-group at P30. **(B)** There is no difference in retinal thickness on P14. **(C)** However, OIR retinae become statistically thinner by P30; retinal thickness is preserved by anti-IL-1β. *N* = 4 animals on P14; *N* = 3-4 on P30. Values are mean ± SEM. One-way ANOVA; ^∗^*p* < 0.05, ^∗∗^*p* < 0.01, ^∗∗∗^*p* < 0.001, ^****^p < 0.0001 vs corresponding values as indicated.

Retinal thickness was defined as that between the IRL and the ORL (Figure 2A). No difference in RT between groups was yet detected immediately after vasoobliteration (during hyperoxia) on P14 (Figure 2B). By P30, a thinner retina was observed in the untreated OIR group, while RT was preserved in OIR animals that received rytvela or Kineret (Figures 2A,C).

### Early Anti-IL-1 β Therapy Reduces the Number of Apoptotic Cells and Preserves Retinal Vessels in the Superficial Capillary Plexus of OIR Animals

**FIGURE 3:**
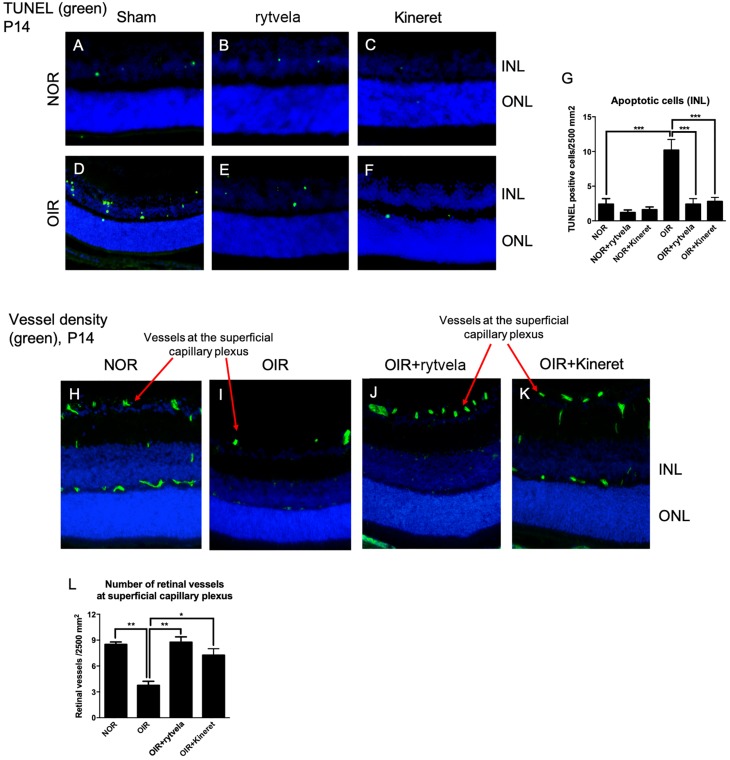
Early anti-IL-1 β therapy reduces the number of apoptotic cells and preserves retinal vessel density at the superficial capillary plexus of OIR subjects. **(A–D)** TUNEL staining show 2–4 positive cells per 2500 mm^2^ in P14 control (NOR) animals (with or without anti-IL-1β), compared to ∼10 cells in OIR rats of the same age. **(E,F)** Both IL-1β antagonists effectively diminish TUNEL positive cells in OIR subjects. Statistical analyses are shown in **G**. **(H)** P14 control animals display normal vessel density (green) in the retina. **(I)** P14 OIR rats show reduced vessel density at the superficial plexus. **(J–L)** Rytvela and Kineret preserved retinal vessels at the superficial capillary plexus of OIR rats. *N* = 3–4 animals per group. Values are mean ± SEM. One-way ANOVA; ^∗^*p* < 0.05, ^∗∗^*p* < 0.01, ^∗∗∗^*p* < 0.001, ^****^p < 0.0001 vs corresponding values as indicated.

Diminished RT in OIR was confirmed histologically (Figure 3); inhibition of IL-1R with rytvela and Kineret avoided retinal thinning. As anticipated, retinal vessel density in the superficial capillary plexus of the nerve fiber layer was reduced by OIR and preserved by anti-IL-1 receptor treatments (Figures 3G,H,I,L). Coherently, microvascular decay which results in the loss of retinal parenchyma was associated with increased apoptosis (TUNEL positivity) mostly observed in the inner retina (Figures 3A,D) consisting of the region most affected in OIR; again, both rytvela and Kineret effectively diminished the number of TUNEL-positive apoptotic cells (Figure 3G).

### Biased Signaling Pathway Selectivity of Rytvela Compared to Kineret

**FIGURE 4:**
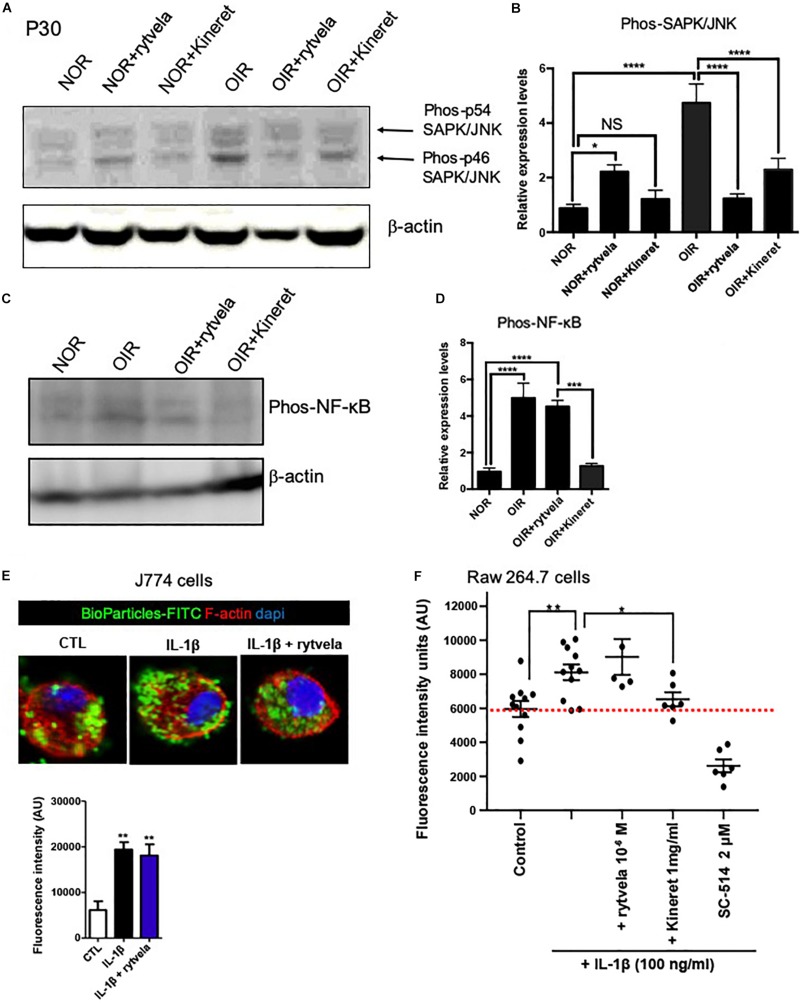
Different inhibition profiles between rytvela and Kineret in retinal tissue at P30 in OIR subjects vs. controls (NOR) and the effect of rytvela on macrophage phagocytosis. **(A,B)** Both rytvela and Kineret abolish SAPK/JNK phosphorylation in OIR subjects. **(C,D)** However, Kineret inhibits NF-κB, whereas rytvela preserves NF-κB pathway. *N* = 3–5 animals. Values are mean ± SEM; ^∗^*p* < 0.05, ^∗∗^*p* < 0.01, ^∗∗∗^*p* < 0.001, ^****^p < 0.0001 vs corresponding values as indicated. **(E)** Confocal imaging showing Fluorescein-labeled BioParticles (green) phagocytosed by J774 mononuclear cells counterstained with Rhodamine phalloidin (red) and DAPI (blue). Histogram below immunofluorescent images refer to quantitative analysis using fluorescence intensity plate reading, showing that rytvela does not inhibit IL-1β-induced phagocytosis. **(F)** Quantitative analysis of Raw 264.7 cell phagocytosis activated by IL-1β, showing preservation by rytvela (1 μM) but not by Kineret (1 mg/ml) or the selective NF-kB inhibitor SC-514 (2 μM). *N* = 4–11/group. One-way ANOVA; ^∗^*p* < 0.05, ^∗∗^*p* < 0.01, ^∗∗∗^*p* < 0.001, ^****^p < 0.0001 compared to control.

Consistent with previous reports (Nadeau-Vallee et al., 2015), rytvela selectively reduced OIR-generated (augmented) SAPK/JNK pathway while preserving intact the important immuno-vigilant related NF-κB axis as measured directly in retinal tissue (at P30) (Figure 4 and Supplementary Material); in comparison, Kineret inhibited both SAPK/JNK and NF-κB pathways (Figures 4C,D). This particularly relevant information results in maintenance of NF-κB-dependent (defense-related) phagocytosis in IL-1β-activated mononuclear myeloid cells by rytvela (Figures 4E,F); whereas phagocytosis in mononuclear cells is compromised by Kineret, as seen with the NF-κB inhibitor SC-514 (Figure 4F).

## Discussion

In this longitudinal study, retinal thinning in OIR animals and the retina-preserving effects of two anti-IL-1β agents, Rytvela and Kineret, were successfully observed using OCT imaging and automated segmentation. Validity of OCT, without established automated segmentation, has been successfully used in assessing retinoblastoma growth (Corson et al., 2014), as well as in evaluating retinal layer injury upon subretinal injections in rats (Becker et al., 2017). The current study extends the reliability of OCT in rodent OIR model. OCT imaging has rapidly become an attractive alternative to current laboratory techniques such as IHC due to its many advantages. First, the traditional IHC to measure RT requires sacrificing animals with significantly longer sample process time. Second, IHC often faces artifacts including tissue shrinkage, swelling, and cracks that are caused by fixation and the postmortem status (Rastogi et al., 2013). In addition, OCT is non-invasive and hence allows longitudinal measurements in the same subject, while conventional IHC protocol requires animal sacrifice at each time point; this translates into a significant reduction of experimental animals needed for longitudinal studies. Lastly, repeated measurements reduce inter-individual variabilities and enhance statistical power. Another strength of this study lies in the use of an automated segmentation protocol which enables timely, rapid, and effective analysis of retinal features, while ensuring objectivity when compared to manual tracing. Accordingly, large amounts of data can be efficiently processed; consistent results can be obtained when repeating iterations on a given dataset, and both inter- and intra-evaluator variabilities are eliminated (Dysli et al., 2015).

A key point to successfully carrying out longitudinal measurements in OCT lies in maintaining a clear cornea in experimental animals. Previous studies have identified a rare but serious side effect of Xylazine (a common anesthetics used in rodent studies)—corneal calcification akin to band keratopathy (Turner and Albassam, 2005; Koehn et al., 2015; Zhou et al., 2017). The dense calcification in the central cornea immediately precluded subsequent OCT experiments. Our group developed a protocol to safely anesthetize animals and achieve long-term, repeated imaging (Zhou et al., 2017). This protocol was applied in our experiments. As expected, results generated by our OCT imaging and automated segmentation are parallel to previous studies that used IHC methods (Dorfman et al., 2008; Dorfman et al., 2010; Rivera et al., 2013); our protocol can thus be readily integrated for reliable longitudinal small animal experimentation.

Anti-IL-1β treatment has been shown to be effective in preventing retinal OIR-elicited vasoobliteration and in turn reduce aberrant pre-retinal neovascularization (Rivera et al., 2013). In this context, it was shown that anti-IL-1 treatment effectively diminishes the release of vaso-repulsive molecule semaphorin 3A from RGCs (Joyal et al., 2014; Sitaras et al., 2015), thereby facilitating NOR revascularization. The advantage of rytvela, a non-competitive inhibitor of IL-1β receptor, has been previously demonstrated in a model of preterm labor (Nadeau-Vallee et al., 2015). In particular, rytvela preserves NF-κB axis while inhibiting SAPK/JNK and others; this effect was shown *in vivo* herein. This is important in maintaining the multiple functions of the transcription factor NF-κB (Hoesel and Schmid, 2013). NF-κB has a quintessential role in sustaining innate immune surveillance (Hayden et al., 2006) consistent with our findings related to mononuclear cell phagocytosis; this is particularly important for premature newborns as they face complex immunological challenges when they emerge from the sterile *in utero* environment (McDonagh et al., 2004; Marchini et al., 2005). During this period, neonates mainly depend on the innate immunity where Toll-like receptor—NF-κB axis plays a significant role (Kollmann et al., 2012). Therefore, when treating neonatal inflammatory conditions, one must strike a fine balance between adequate immune defense and unrestrained inflammation. Additionally, NF-κB signaling participates in cell proliferation and angiogenesis in the retina (Yoshida et al., 1999; Noort et al., 2014). In ROP, revascularization and restoration of retinal blood flow are prerequisites for the proper development of the premature retina (Wei et al., 2016). Overt inhibition of NF-κB by Kineret may contribute to the absence of revascularization in OIR rats as observed herein. Hence together, rytvela provides a beneficial alternative to Kineret as anti-IL-1β treatment of the neonate.

In summary, OCT imaging coupled with an automated segmentation algorithm represents a non-invasive, reliable, and readily efficient method to study longitudinally retinal pathology and its alteration by drug candidates, in rat OIR model. The present study further establishes that rytvela, a novel biased inhibitor of IL-1β, preserves retinal integrity and restores vascular density in a rodent model of ischemic retinopathy, while conserving innate mononuclear cell phagocytosis.

## Data Availability Statement

All datasets analyzed for this study are included in the article/Supplementary Material.

## Ethics Statement

The animal study was reviewed and approved by the Hôpital Maisonneuve-Rosemont Animal Care Committee.

## Author Contributions

DS, TZ, SaC, and SyC contributed to the conception and design of the study. DS, TZ, SO, and CQ carried out the experiments. JM developed the segmentation algorithm. DS and TZ performed the data analyses. DS and TZ wrote the first draft of the manuscript. All authors (including MW, FC, RD, and MD) contributed to interpretation and analysis of data. SaC and SyC provided expert advice and gave important suggestions for improving the manuscript. All authors contributed to manuscript revision and read and approved the submitted version.

## Conflict of Interest

The authors declare that the research was conducted in the absence of any commercial or financial relationships that could be construed as a potential conflict of interest.

## Abbreviations

ANOVA: analysis of variance
ART: automatic real time
Iba-1: ionized calcium-binding adaptor molecule 1
IHC: immunohistochemistry
IL-1: interleukin-1
IL-1Ra: interleukin-1 receptor antagonist
IRL: innermost retinal layer
NOR: normal
P14: postnatal day 14
P30: postnatal day 30
OCT: optical coherence tomography
OIR: oxygen-induced retinopathy
ORL: outermost retinal layer
RGC: retinal ganglion cell
RT: retinal thickness
SD-OCT: spectral domain optical coherence tomography
TUNEL: terminal deoxynucleotidyl transferase dUTP nick end labeling
