# Supplementary figures and images for: Novel Anti-Interleukin-1β Therapy Preserves Retinal Integrity: A Longitudinal Investigation Using OCT Imaging and Automated Retinal Segmentation in Small Rodents
Source: Front Pharmacol. 2020 Mar 12;11:296. doi: 10.3389/fphar.2020.00296 (PMC7081735; doi:10.3389/fphar.2020.00296)

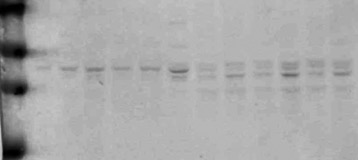

Supplement: Supplementary file 1 [file Data_Sheet_1.zip › Fig4A_phosSAPK_JNK.jpg]

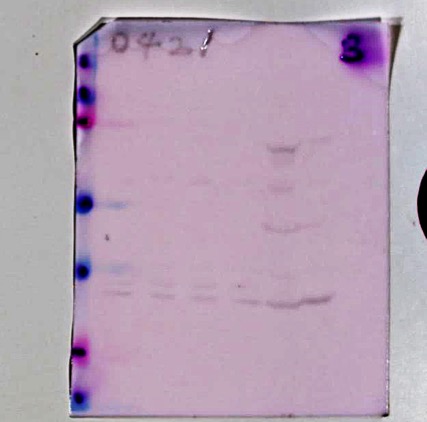

Supplement: Supplementary file 1 [file Data_Sheet_1.zip › Fig4B_bactin.jpg]

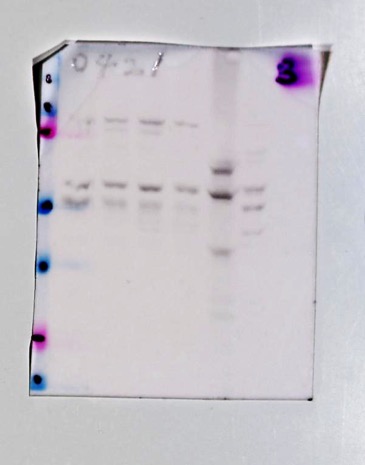

Supplement: Supplementary file 1 [file Data_Sheet_1.zip › Fig4B_phosNFkB.jpg]

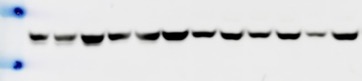

Supplement: Supplementary file 1 [file Data_Sheet_1.zip › Fig4A_bactin.jpg]
