# Supplementary material for: Novel Anti-Interleukin-1β Therapy Preserves Retinal Integrity: A Longitudinal Investigation Using OCT Imaging and Automated Retinal Segmentation in Small Rodents
Source: Front Pharmacol. 2020 Mar 12;11:296. doi: 10.3389/fphar.2020.00296 (PMC7081735; doi:10.3389/fphar.2020.00296)
Supplement: Supplementary file 2 [file Presentation_1.pptx]

## Slide 1
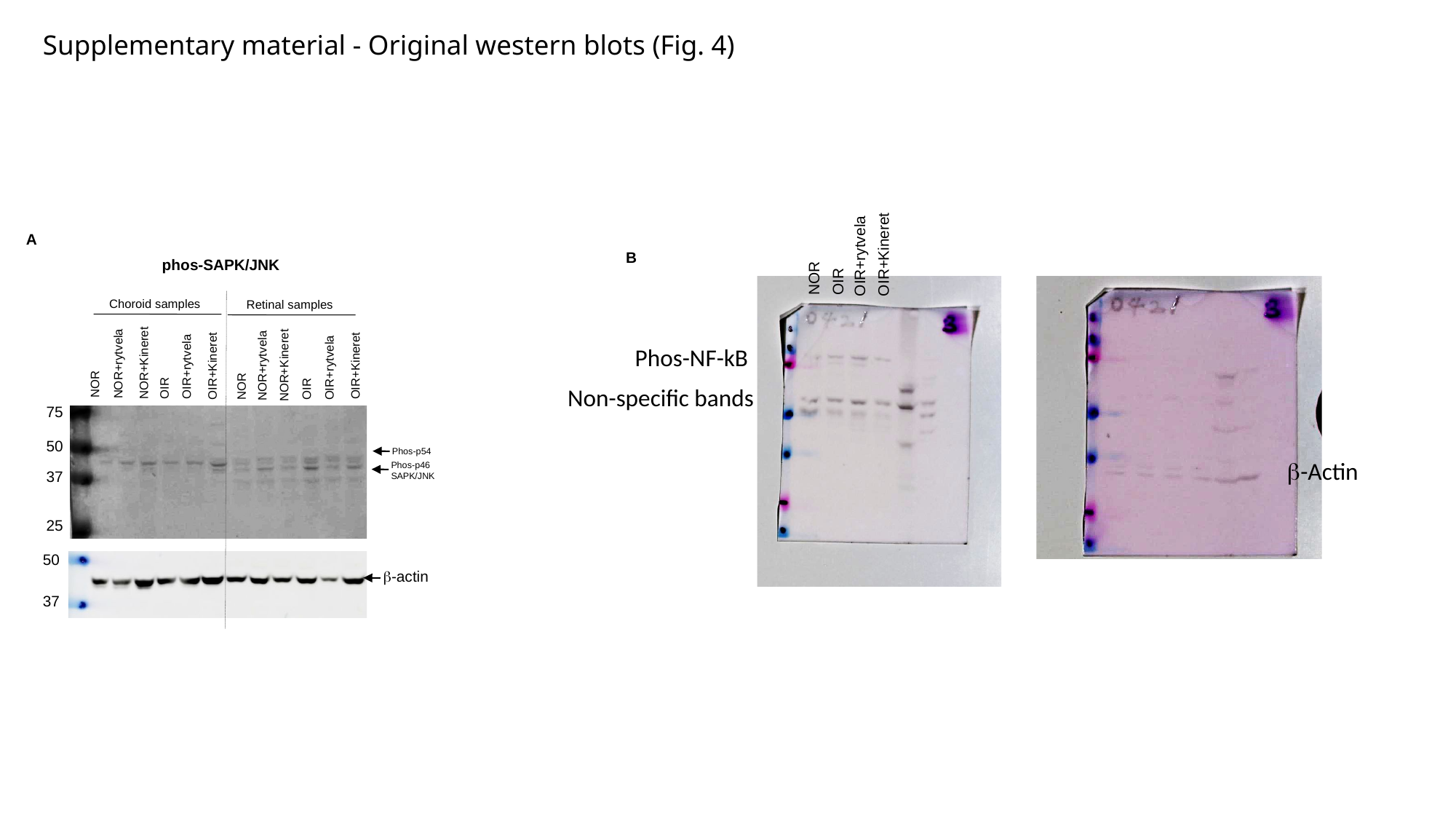

# Supplementary material - Original western blots (Fig. 4)
A
OIR+Kineret
OIR+rytvela
B
phos-SAPK/JNK
NOR
OIR
Choroid samples
Retinal samples
Phos-NF-kB
NOR+Kineret
NOR+rytvela
NOR+Kineret
NOR+rytvela
OIR+Kineret
OIR+Kineret
OIR+rytvela
OIR+rytvela
NOR
NOR
OIR
OIR
Non-specific bands
75
50
Phos-p54
Phos-p46
SAPK/JNK
β-Actin
37
25
50
b-actin
37
